# PhyliCS: a Python library to explore scCNA data and quantify spatial tumor heterogeneity

**DOI:** 10.1186/s12859-021-04277-3

**Published:** 2021-07-03

**Authors:** Marilisa Montemurro, Elena Grassi, Carmelo Gabriele Pizzino, Andrea Bertotti, Elisa Ficarra, Gianvito Urgese

**Affiliations:** 1 Department of Control and Computer Science, Politecnico di Torino, C.so Duca degli Abruzzi 24, 10129 Turin, Italy; 2 Interuniversity Department of Regional and Urban Studies and Planning, Politecnico di Torino, C.so Duca degli Abruzzi 24, 10129 Turin, Italy; 3 Department of Oncology, University of Torino, Strada Provinciale, 142 - KM 3.95, 10060 Candiolo, Turin, Italy; 4 Candiolo Cancer Institute - FPO IRCCS, Strada Provinciale, 142 - KM 3.95, 10060 Candiolo, TO Italy; 5 Enzo Ferrari Engineering Dept, University of Modena and Reggio Emilia, Via Vivarelli 10/1, 41125 Modena, Italy

**Keywords:** Single-cell sequencing, Intra-tumor heterogeneity, Cancer evolution, Clones, DNA, Algorithms

## Abstract

**Background:**

Tumors are composed by a number of cancer cell subpopulations (subclones), characterized by a distinguishable set of mutations. This phenomenon, known as intra-tumor heterogeneity (ITH), may be studied using Copy Number Aberrations (CNAs). Nowadays ITH can be assessed at the highest possible resolution using single-cell DNA (scDNA) sequencing technology. Additionally, single-cell CNA (scCNA) profiles from multiple samples of the same tumor can in principle be exploited to study the spatial distribution of subclones within a tumor mass. However, since the technology required to generate large scDNA sequencing datasets is relatively recent, dedicated analytical approaches are still lacking.

**Results:**

We present PhyliCS, the first tool which exploits scCNA data from multiple samples from the same tumor to estimate whether the different clones of a tumor are well mixed or spatially separated. Starting from the CNA data produced with third party instruments, it computes a score, the Spatial Heterogeneity score, aimed at distinguishing spatially intermixed cell populations from spatially segregated ones. Additionally, it provides functionalities to facilitate scDNA analysis, such as feature selection and dimensionality reduction methods, visualization tools and a flexible clustering module.

**Conclusions:**

PhyliCS represents a valuable instrument to explore the extent of spatial heterogeneity in multi-regional tumour sampling, exploiting the potential of scCNA data.

**Supplementary Information:**

The online version contains supplementary material available at 10.1186/s12859-021-04277-3.

## Background

Tumors are caused by the accumulation of somatic mutations. The set of mutations accumulated by the founder cell of a tumor is defined as clonal and inherited by its entire progeny. The mutations arising in an already existing tumor are passed on only to sub-populations of cells and are defined as subclonal [1, 2]. As a result, cancer cells are characterized by an intrinsic genetic diversity, known as intra-tumor heterogeneity (ITH) [3].

ITH is a major topic of interest for the cancer research community, since it has been recognized as one of the major responsible for tumor relapse and treatment failure [3–7]. The most common way to assess ITH is to use deconvolution techniques on bulk DNA sequencing data [8, 9]. Such techniques are generally based on machine learning models, used to cluster the mutations into subclones based on their prevalence and exploit such clusters to infer the tumor phylogenetic structure [10–18]. Some studies have proposed methods to evaluate ITH based on gene expression [19–21] or protein-protein interactions [22].

Several studies have shown that using multiple samples taken from distinct regions of the same lesion improves the ability to infer the subclonal structure of tumors [3–5, 23–28] and assess ITH. For example, a study conducted by Jamal-Hanjani et al. [29], sampling 327 regions from 100 early stage non-small-cell lung cancers, revealed that 30% of the somatic mutations were subclonal and stated that if fewer regions had been sampled, many of those mutations would have misinterpreted as clonal.

In this context, emerging single-cell DNA sequencing (scDNA-seq) technologies offer an extraordinary opportunity to tackle such issues, as they allow to study tumor heterogeneity with unprecedented resolution. In particular, single-cell low-coverage whole genome sequencing is suited for detecting chromosomal aberrations, which can be exploited to reconstruct cell population subclonal structure [30].

However, the existing methods for single-cell CNA (scCNA) analysis are still limited. Many of them [31–38] only identify the total copy-number, which indicates the sum of the number of copies at each locus, by analyzing differences between the observed and expected number of sequences aligned to a locus, or the read-depth ratio. A few of them, also, infer the tumor phylogeny using the CNAs they computed [39].

However, to our knowledge, an instrument capable of exploiting both the granularity of single-cell DNA data and multi-sample analysis to quantify ITH still does not exist.

Therefore we present PhyliCS, a flexible Python library that explores CNA calls obtained with third-party tools and exploits them to compute a new metric, the Spatial-Heterogeneity score (SHscore). This score is useful to evaluate the spatial heterogeneity of tumors when multiple regional samplings are available, quantifying how much cells from different samples from the same patient have diverged in their CN landscapes. This evaluation allows both to rank different tumors based on their heterogeneity and identify the most divergent spatial samples of a given tumor. Additionally, it may help to explore different tumors without a huge number of sequenced cells and/or regional samplings to select only the most heterogeneous ones for further analyses.

Moreover, PhyliCS provides easy access to several clustering methods for both single and multiple samples to users, making it easy to compare results and tailor each analysis to each specific experiment. We show its potential by running it on 300 simulated datasets, to validate the SHscore on some selected ideal scenarios where it compares sets of cells with known relationships. After that, we demonstrate the correlation between the proposed SHscore and the evolutionary distance between the cells of the samples in analysis, through a more extensive simulation experiment. Lastly, we present the results of the analysis on three publicly available scDNA datasets, one with multiple spatial samplings from a breast tumor, another comprised of a primary lung tumor and its derived metastases and a third one with a cell line and two clonal expansions of two single cells, using the SHscore to describe how their CN profiles differ when considering the fine grained single cell level in the bigger context of multiple sampling.

### Implementation

In this section we will first describe the main modules of PhyliCS; then we will present the mathematical details of the SHscore and its interpretation.

#### PhyliCS

PhyliCS is a comprehensive toolkit that integrates scCNA calls analysis procedures into a single and modular Python package.

**Fig. 1:**
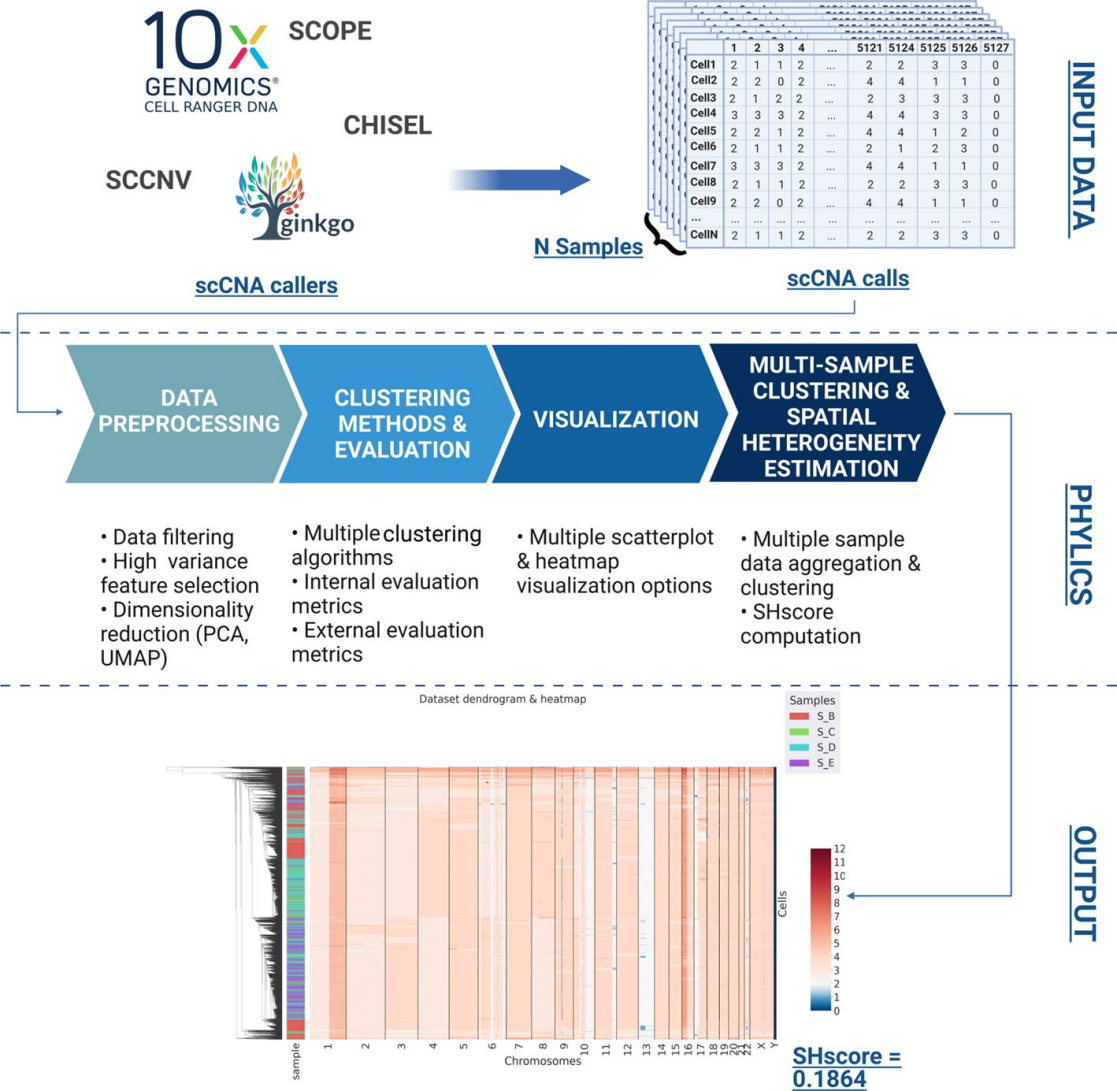
PhyliCS logical schema. PhyliCS allows to perform downstream analysis on the scCNA profiles computed with scCNA third party callers. Specifically, it accepts tabular data and allows to perform data filtering, feature selection, dimensionality reduction, to prepare the data before executing one of the multiple available clustering algorithms. It also allows to perform clustering result quality evaluation by means of both internal and external evaluation metrics. But, most importantly, it provides the possibility to aggregrate scCNA data from multiple samples, to jointly cluster and visualize them, estimating their spatial ITH through the SHscore

As Fig. 1 shows, PhyliCS takes as input the scCNA calls produced by one of the existing scCNA callers [31–39] and allows the users to perform:

- data preprocessing (feature selection, PCA, UMAP, data filtering),
- data visualization (UMAP-based scatterplots, heatmaps),
- data clustering (Affinity Propagation [40], Birch [41], DBSCAN [42], HDBSCAN [43], Hierarchical Agglomerative [44], KMeans [45], OPTICS [46], Spectral [47]),
- clustering algorithms evaluation (Silhouette Coefficient, Davies-Bouldin Index, Calinski-Harabasz Index, Adjusted Rand Index, V-Measure, Fowlkes-Mallows Score, Mutual Information),
- multi-sample clustering, visualization and spatial intra-tumor heterogeneity estimation (SHscore).

PhyliCS multi-sample analysis module works on the aggregation of input sample data and produces two main results: a graphical representation and a numerical quantification of spatial intra-tumor heterogeneity, the SHscore. Specifically, it generates an aggregated heatmap with a dendrogram computed performing hierarchical clustering of the cells. Heatmap rows, representing the cells from the different samples, are identified by different colored labels. In this way, it is possible to assess whether the clustering algorithm segregated cells originating from different samples into different branches of the dendrogram or if generated mixed clusters: the former case would indicate that, despite originating from the same tumor, the genomic make-up of the cells belonging to different samples is different (spatial intra-tumor heterogeneity); the latter case, instead, would denote that different samples are populated by cells with a similar genomic variance.

PhyliCS implementation is based on a dedicated class, named *CnvData*, which is a modular data structure storing all data annotations (e.g. cell ploidy, cell MAD, etc.) and the results of each analytical step (e.g. PCA, clustering results, etc.) without affecting the data matrix. On the one side, this implementation choice simplifies and speeds up computation; on the other side, it allows experienced developers to extend the framework and add new functionalities with a low programming effort.

PhyliCS does not represent an alternative to the existing scCNA tools developed for identifying scCNA events [31–38] or tools designed for the phylogenetic analysis [39]. Indeed, PhyliCS offers an API to work on scCNA data, leveraging outputs of different third-party tools, and implements a method to characterize spatial ITH.

#### Spatial Heterogeity Score

The Spatial-Heterogeneity score (SHscore) is a relative measure of how much the genomic make-up of different samples taken from the same patient diverges with respect to the internal variance of each sample.

**Fig. 2:**
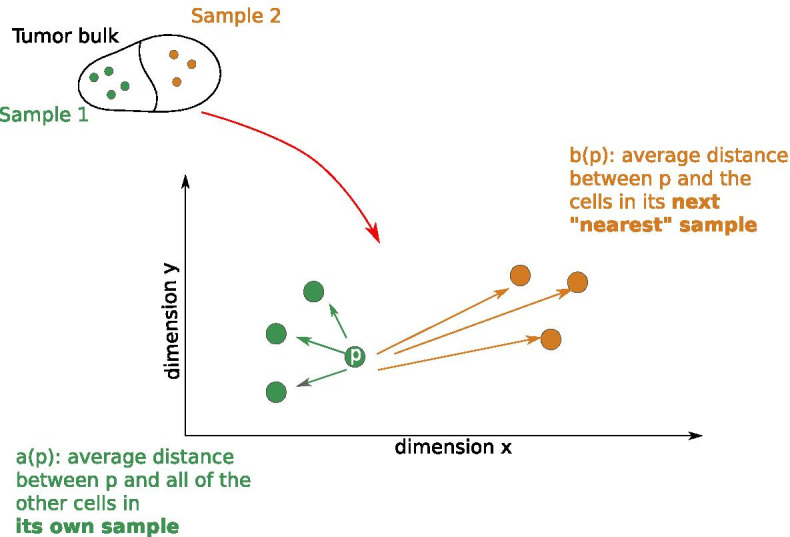
Intra and inter-sample pairwise distance. Given the cell *p*, *a*(*p*), is the average pairwise distance between *p* and all other cells from its own sample, while *b*(*p*) is the average pairwise distance between *p* and the cells from the “nearest” sample

*Definition* The principles underlying the SHscore are inspired to those of the Silhouette score, an index used in classical Data Science, to estimate the quality clustering results [48]. In fact, we can think of cells as data-points, described by their CNA profile, and of the samples as the cluster they belong to. It is possible to compute for each cell, *p*, the average distance from all other cells belonging to its own cluster, *a*(*p*), and then compare it to the average distance from the cells belonging to the “nearest”, or most similar, cluster, *b*(*p*). Figure 2 shows a conceptual schema of a tumor divided into two subsamples: green arrows represent the pairwise distance between a given cell, *p*, and all cells of its sample; the orange ones, the distance between the same cell and cells of the nearest sample. The average computed on these distances are *a*(*p*) and *b*(*p*).

These distances are the same used to compute the Silhouette score, so we can re-use its implementation and adapt it for our purposes.

For each cell *p* and sample $S_p$, such that $p \in S_p$, let *a*(*p*) (Eq. 1) be the average pairwise-distance between *p* and the other cells belonging to its sample and *b*(*p*) (Eq. 2) be the minimum average pairwise-distance between *p* and other sample cells. Now, we can compute *sh*(*p*) (Eq. 3) which measures the difference between the average pairwise-distance between *p* and the cells of the sample, nearest to the one it belongs to, and the average pairwise-distance between *p* and the cells of its own sample.

1 $$\begin{aligned} a(p)= & {} \frac{1}{\mid S_p \mid - 1}\sum _{p \in S_p, q \ne p}d(p,q) \end{aligned}$$

2 $$\begin{aligned} b(p)= & {} min_{k \ne p}\frac{1}{\mid S_k \mid }\sum _{q \in S_k} d(p,q) \end{aligned}$$

3 $$\begin{aligned} sh(p)= & {} \frac{b(p) - a(p)}{max\{a(p), b(p)\}} \end{aligned}$$

Dividing it by $max\{a(p), b(p)\}$ makes *sh*(*p*) a relative difference.

In order to mitigate the negative impact of the high dimensionality of scCNA data, we adopted *L1*, or *Manhattan*, norm to compute pairwise distances. In fact, it has been demonstrated that, for dimensionalities of 20 or higher, *LK* norms, with $K \le 1$, better discriminate [49, 50] between the nearest and the furthest neighbors compared to higher level norms (e.g. *L2*, or *Euclidean* norm).

From Eq. 3 it is clear that $-1 \le sh(p) \le +1$.

For *sh*(*i*) to be close to 1 we require $a(p)<< b(p)$. As *a*(*p*) is a measure of how much the genomic profile of *p* is dissimilar to the average profile of its own sample, a small value means a high level of similarity. Furthermore, a large *b*(*p*) indicates that *p* CNA profile is highly different from the average profile of the most similar among the samples in analysis. Thus, a *sh*(*p*) close to 1 means that *p* CNA profile matches the average genomic profile of the sample it belongs to. If *sh*(*p*) is close to −1, then by the same logic we can state that *p* CNA profile is more similar to the genomic profile of the neighboring sample than to the genomic profile of the other cells of its own sample. An *sh*(*p*) close to 0 means that the CNA profile is on the border of two natural clusters, so *p* may belong to both of them.

Mathematically, the SHscore, $SHscore(S_1, S_2, \ldots , S_n)$, for the set of samples $S_1, S_2, \ldots , S_n$, is a measure of how well-separated the samples are and is defined as the mean *sh*(*p*) over all cells in the entire dataset, $D = [S_1 \cup S_2 \cup \cdots \cup S_n]$ (Eq. 4).

4 $$\begin{aligned} SHscore(S_1, S_2, \ldots , S_n) = \frac{\sum _{p, p \in D} sh(p)}{\mid D \mid }. \end{aligned}$$

From Eq. 4, it is clear that also the SHscore may assume values in the interval $[-1, 1]$ and its interpretation may be derived from the interpretation of single-cell scores. Specifically, a SHscore close to 1 indicates that many cells, in the various samples, are characterized by a *sh*(*p*) close to 1, denoting that samples are internally homogeneous and segregated with respect to the others. Similarly, a SHscore close to −1 indicates that many cells, in the dataset, look more similar to the cells of another sample than to those of their own sample; this, could denote problems with the sequencing quality or data pre-processing. Finally, a SHscore close to 0 implies that many cells may indistinctly belong to their own sample or to another, which may indicate two scenarios: the samples are internally homogeneous, but very similar among each other, thus they share the same subclonal structure and cells may belong to one or another; or that the samples are internally heterogeneous, so that the CN profiles of their cells cannot be clearly assigned to any one of them.

*Application scenario*

Let us suppose that three single-cell data-sets, $s_1, s_2, s_3$, originated from three different regions of the same tumor, have been provided as input samples to PhyliCS. The SHscore evaluation phase will proceed as follows:

1. The cells are assigned to three predefined clusters, $S_1, S_2, S_3$, in the following way: $\{p : p \in s_i\} \Rightarrow p \in S_i$, where $i \in [1,2,3]$. The SHscore is computed as $hs_{1,2,3} = SHscore(S_1, S_2, S_3)$
2. The cells from $s_1$ and $s_2$ are combined in a single cluster, $S_{12}$, and those from $s_3$ are assigned to a separate cluster, $S_3$. The SHscore is computed again as $hs_{12,3} = SHscore(S_{12}, S_3)$.
3. The cells from $s_1$ and $s_3$ are combined in a single cluster, $S_{13}$, and those from $s_2$ are assigned to a separate cluster, $S_2$. The SHscore is computed again as $sh_{13,2} = SHscore(S_{13}, S_2)$
4. The cells from $s_2$ and $s_3$ are combined in a single cluster, $S_{23}$, and those from $s_1$ are assigned to a separate cluster, $S_1$. The SHscore is computed again as $sh_{23,1} = SHscore(S_{23}, S_1)$.

Let us suppose, now, that $hs_{23,1}$ is the maximum computed score. Specifically, we suppose that:

5 $$\begin{aligned} sh_{23,1} > sh_{1,2,3}. \end{aligned}$$

This means that samples $S_2$ and $S_3$ are similar to each other and, in some measure, different from sample $S_1$ and that considering their cells together resulted in a better clustering.

To conclude, the SHscore represents a way to quantify numerically the genomic distance, in terms of CNAs, between different samples of the same tumor and to investigate spatial intra-tumor heterogeneity.

## Results and Discussion

Here, the experiments conducted to study the SHscore behaviour in different contexts are introduced. Additionally, the procedures executed to generate the simulated datasets are described.

In details, the SHscore has been used on 200 simulated datasets representing some ideal scenarios (spatial segregation, spatial intermixing, early metastasis spreading and late metastasis spreading), to check if it correctly reflects the heterogeneity in the clonal structure of multiple samples. After that, the score has been tested on a set of 100 simulations to analyze its behaviour when the mean CNA size and the mean number of copies gained varies in a controlled way. Then, a more extensive simulation was conducted to verify the correlation between the SHscore and the divergence accumulated during the evolution of the samples. Finally, the SHScore has been tested on 3 publicly available scCNA datasets to study its behaviour in some real-world scenarios.

### Experiment 1: SHscore on synthetic data

#### Data generation

We conducted a simulation study to analyze the SHscore behaviour under four different scenarios (spatial subclone segregation, spatial subclone intermixing, early and late metastasis spreading) and to study if and how it correlates with some features of the CN profiles of cells (CNA region size, CN level).

To this purpose, we extended the model presented by Fan et al. [51] which generates a phylogenetic tree starting from a reference genome, using a generalization of the Beta-Splitting model [52]. At the end of the simulation process, the leaves of the generated tree represent the cells sampled from the patient, while the internal nodes represent intermediate CN states, which do not exist anymore.

**Fig. 3:**
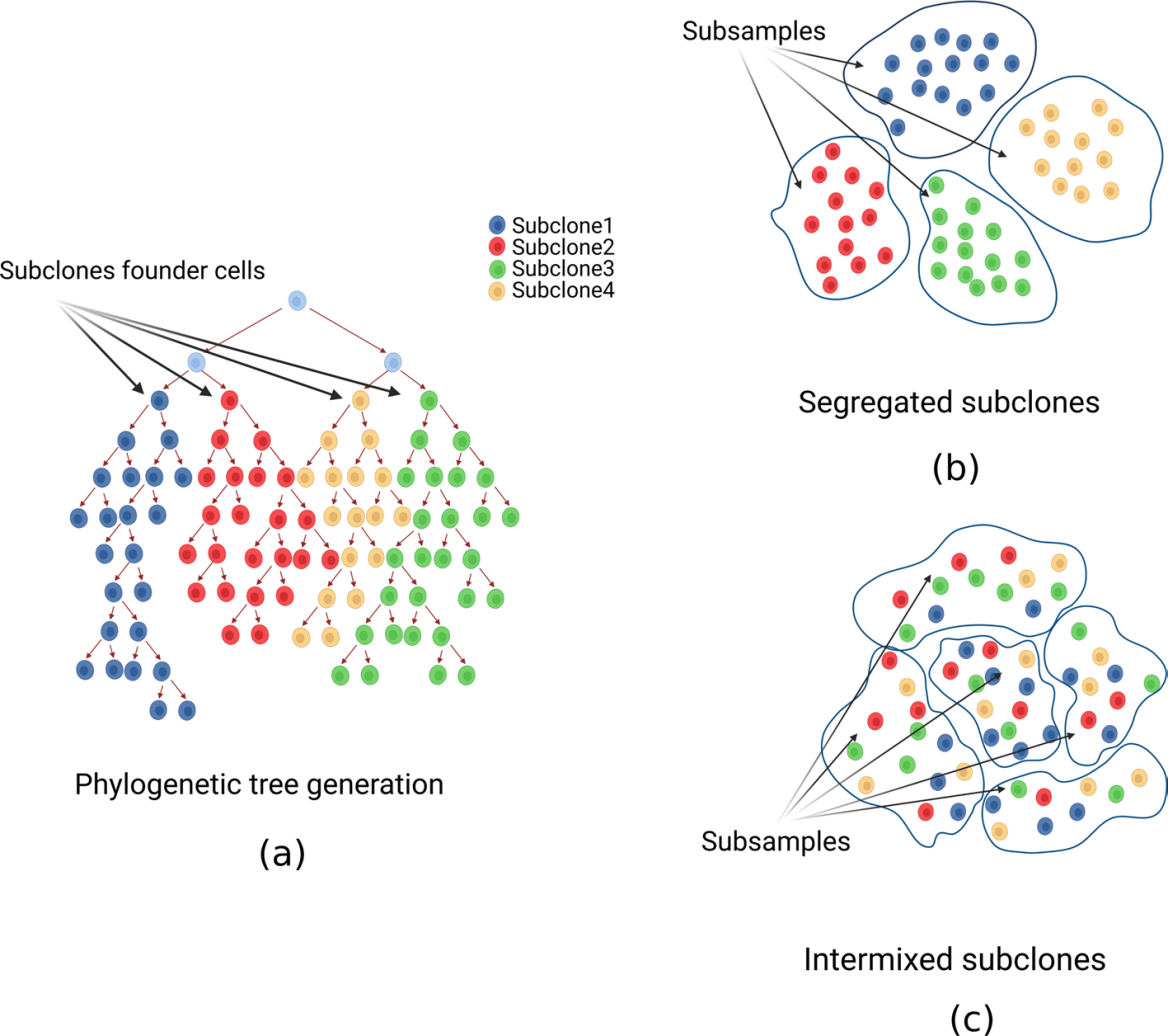
Spatial subclonal segregation and intermixing simulation. We generated 50 phylogenetic trees (**a**) made of 2500 cells. For each tree we simulatated: (I) early segregation of subclones (*hom*) by tracking the progeny of the first five generated cells and assigned the leaves to five distinct subsamples, corresponding to the five subclones (**b**); (II) spatial intermixing of subclones (*het*) by shuffling the leaves and assigning them randomly to five subsamples (**c**)

*Spatial segregation* To simulate the extreme case in which subclones segregate in isolated niches very early during tumor evolution, we tracked the progeny of the first 5 cells (Fig. 3a) generated by the simulator. We let the trees grow until they contained 2500 leaves. At that point, we were able to distinguish groups of cells phylogenetically separated and to consider them as our subsamples, each containing a distinct subclone (Fig. 3b). So, in the end, we divided each dataset into 5 subsamples corresponding to the 5 groups of cells deriving from the first 5 generated cell. From now on, we refer to this scenario as *hom-scenario*.

*Spatial intermixing* We also simulated the scenario in which the tumor cells subpopulations are spatially well-mixed, so a regional subsampling would produce very similar samples. This was done by shuffling the leaves of the previously generated trees and randomly assigned them to 5 subsamples (Fig. 3c). From now on, we refer to this scenario as *het-scenario*.

**Fig. 4:**
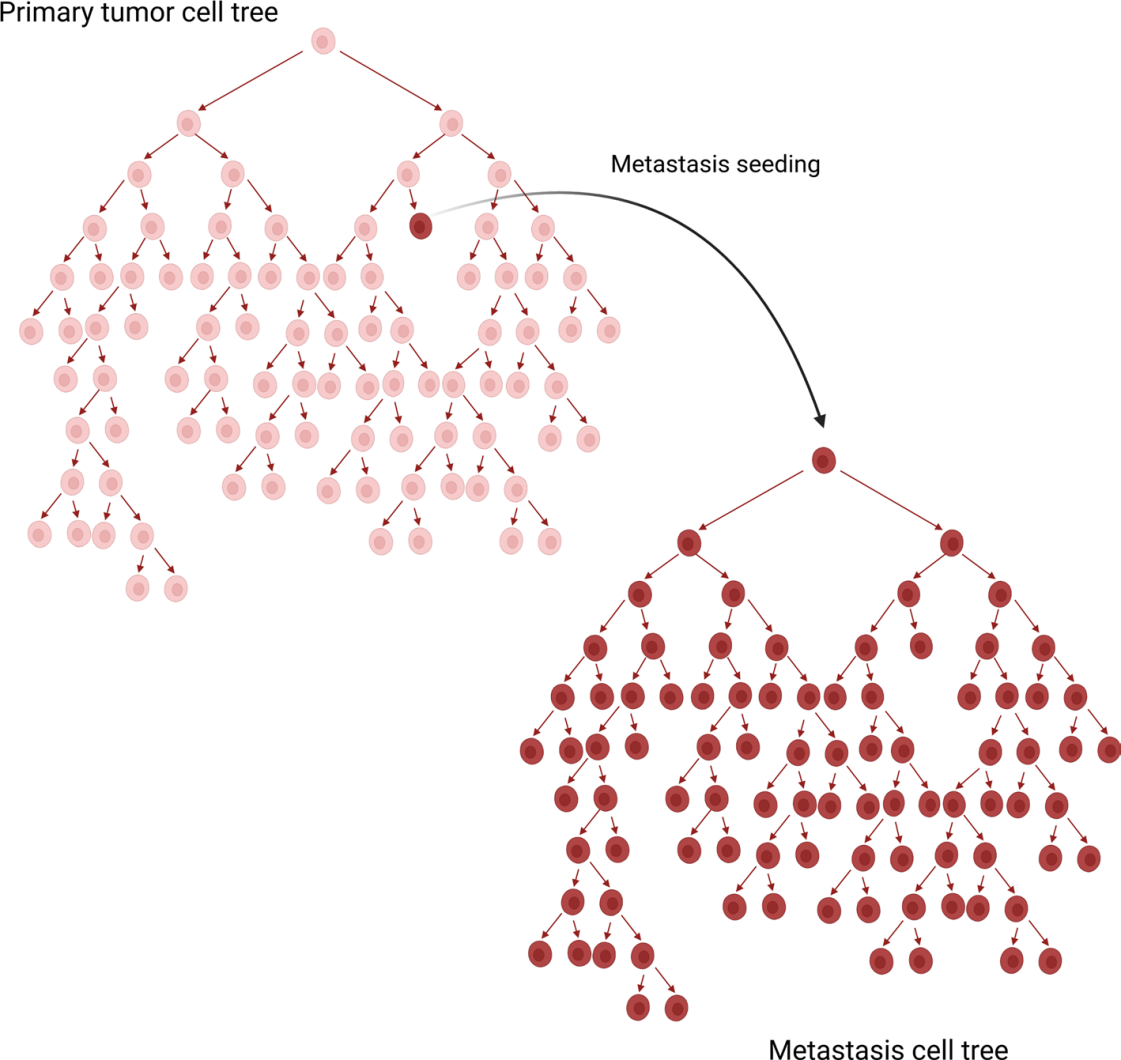
Metastasis seeding and expansion simulation. We generated 100 pairs of primary-metastasis samples (50 *early* metastasising, 50 *late* metastasising). Each pair was obtained by seeding the primary tumor tree and successively initiating a new tree with a cell randomly selected when the primary tree had generated 1/3 (early) or 3/4 (late) of the final number of desired cells. The simulation was stopped when both trees had generated 500 leaves

*Metastasis spreading* We simulated another different case of spatial segregation, which is the scenario in which a cell seeds a metastasis, initiating a completely isolated clonal expansion. To that purpose we generated new phylogenetic trees: when the trees had generated 1/4 or 3/4 of the final number of cells, we randomly selected one cell and seeded another tree to model early or late metastatic spreading during the primary tumor evolution, respectively. We let the tree generation proceed in parallel until all of them contained 500 leaves (Fig. 4). From now on, we refer to these scenarios, respectively, as *early-met-scenario* and *late-met-scenario*.

For each of the four scenarios described so far, we generated 50 synthetic datasets for a total of 200 simulations.

*Simulations with varying parameters* 100 datasets were simulated with varying parameters to generate CN profiles characterized by different structural features and check if and how those features correlate to the SHscore. Precisely, we varied the expected CNA size ($\theta$), which is used by the simulator to sample from an exponential distribution, and the reciprocal of the expected number of gained copies (*p*), which is used to sample from a geometrical distribution. In details, for each simulation, $\theta$ was chosen by randomly sampling from a uniform distribution defined in the interval [500, 5000000], while *p* was sampled from a uniform distribution defined in the interval [0.1, 0.9] (Supplementary Material: Supplementary Figures 1a and 1b). Each simulated tree had 1000 leaves and was splitted into two subtrees, each representing a tumor subsample. From now on, we refer to this scenario as *var-scenario*.

#### SHscore statistics

SHscore was computed on the synthetic datasets, built to represent the previously described heterogeneity scenarios, to evaluate its ability to capture their differences.

**Fig. 5:**
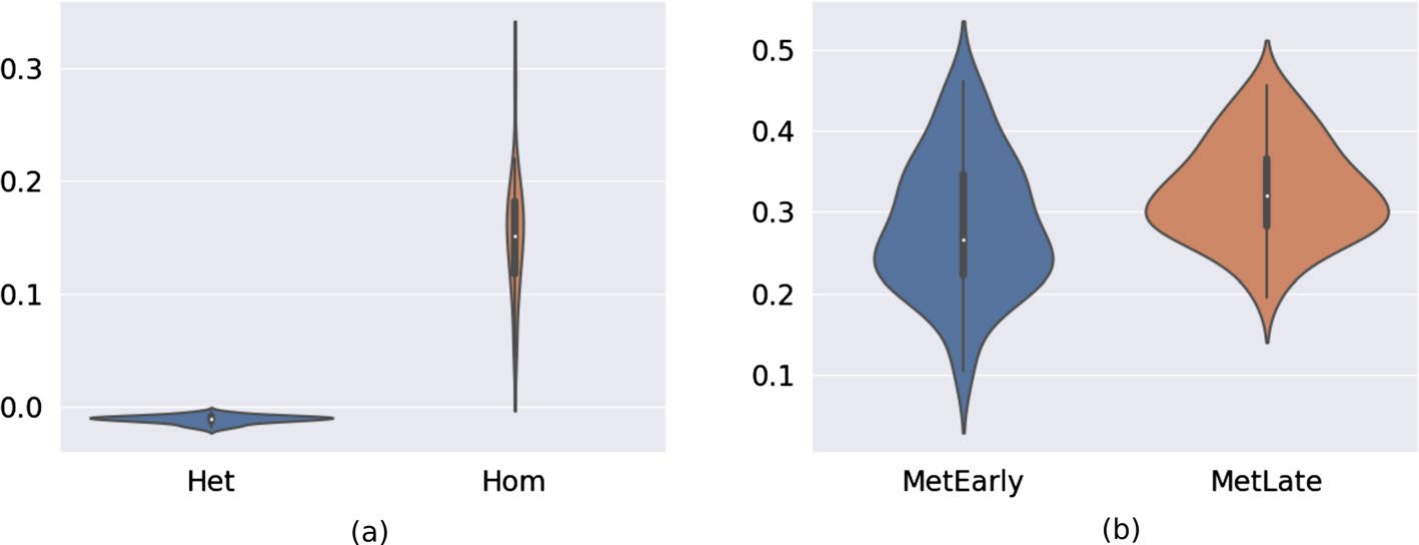
SHscore distribution. We computed the SHscore on 100 synthetic datasets simulating regional subsampling (**a**) (Mann–Whitney U test p-value $3.5 \times 10^{-18}$). Het-scenario =  min: -0.020, max: -0.004, median: -0.010, IQR: 0.004. Hom-scenario = min: 0.043, max: 0.295, median: 0.151, IQR: 0.064. We also computed the SHscore on 100 synthetic dataset simulating metastasis spreading (**b**)(Mann–Whitney U p-value 0.0029). EarlyMet scenario = min: 0.103, max: 0.461, median: 0.267, IQR: 0.124; LateMet scenario = min: 0.195, max: 0.547, median: 0.320, IQR: 0.084

*Spatial heterogeneity at the same disease site* First, we computed the SHscore on the 100 sets of samples simulating the regional subsampling from the same disease site (Fig. 3b, c). Figure 5a shows the SHscores computed on the *hom-scenario* (spatial segregation) and the *het-scenario* (intermixing). The scores, in the two scenarios, are different (unpaired wilcoxon p value $3.5 \times 10^{-18}$): in the *het-scenario* values fall into a very small interval (min: -0.020, max: -0.004, median: -0.010, IQR: 0.004); the *hom-scenario*, instead, produced scores ranging on a broader interval (min: 0.043, max: 0.295, median: 0.151, IQR: 0.064), reflecting a higher heterogeneity between the simulated samples with different “clones” (the progenies of the first five cells) evenly distributed among them (Fig. 5b).

The results obtained by this experiment demonstrated that our score is able to discriminate between the two described scenarios.

*Spatial heterogeneity at different disease sites* Figure 5b shows the results for the two metastatic scenarios: here too the difference is significant (Mann–Whitney U p value 0.0029), albeit less pronounced, underlying how different seeding histories can result in different SHscores; even if with the parameters chosen for our simulations the difference is small and the intra-scenario variability between different simulation is high (*early-met*: min: 0.103, max: 0.461, median: 0.267, IQR: 0.124; *late-met*: min: 0.195, max: 0.547, median: 0.320, IQR: 0.084).

**Fig. 6:**
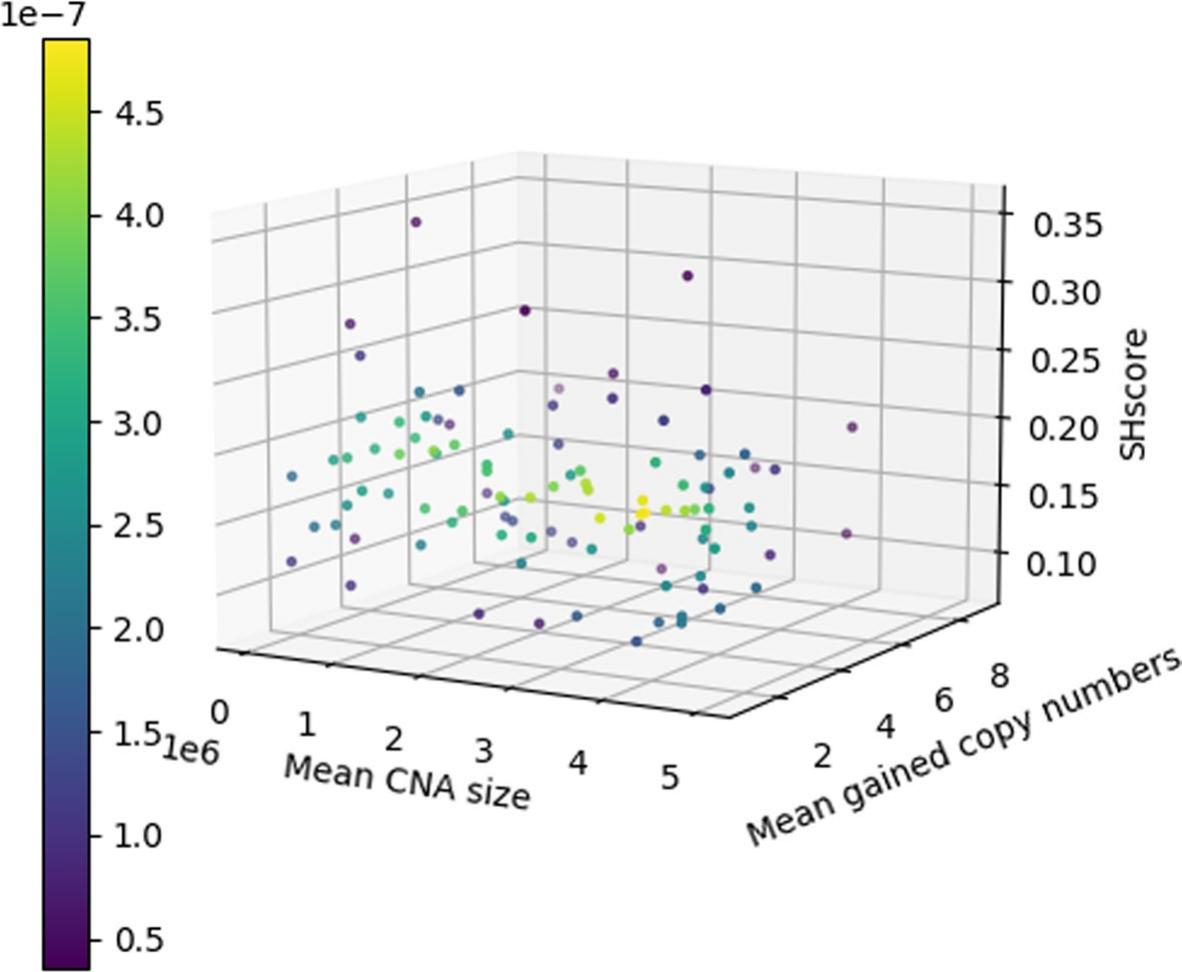
SHscore independence from mean CNA size and mean gained copies. We tested the SHscore on multiple simulated sample pairs, characterized by a different and known mean CNA size $\theta$ and mean number of gained copies *p*. We found out that the SHscore is uncorrelated to those features with a Pearson correlation coefficient c = −0.101 (p value =  0.319), for the mean CNA size, and c = −0.109 (p value = 0.282), for the mean number of gained copies

*SHscore indipendence from CNA size and gained copy number* In order to study if the SHscore correlates with the mean CNA size and the mean number of gained copies, we computed the SHscore for each pair of samples generated in the *var-scenario*. Then, we calculated the Pearson correlation coefficient between the SHscores and the parameters $\theta$ (mean CNA size) and *p* (reciprocal of mean number of gained copies), for each simulation. The results ($\theta$: Pearson correlation coefficient = −0.101, p value = 0.319; *p*: Pearson correlation coefficient = −0.109, p value = 0.282), indicated that there were no significant correlations, suggesting that SHscore is robust with respect to different rates of CN accumulation and to the size of events (Fig. 6).

### Experiment 2: SHscore and evolutionary distance

The heterogeneity quantified by the SHscore reflects the evolutionary distance between the cells of the samples analyzed. Another simulation experiment was designed to verify the existence of a correlation between SHscore and the distance between the copy-number states which originated the mutational profile of the samples. Such CN states may be thought as the most recent common ancestor (MRCA) of the existing CN profiles.

#### Data generation

*100Kcells and 10Kcells.* In order to generate a deep evolutionary history and, consequently, a more heterogeneous dataset, a cell-division tree with 100K final leaves was simulated. The subtrees rooted in the first 200 generated cells were tracked, simulating the complete spatial segregation of the subclones originating from those cells (see Spatial heterogeneity at the same disease site). The cardinality of the generated datasets was quite homogeneous (mean cell number = 500 cells) with some exceptions (min cell number = 91, max cell number = 3112). In order to have a balanced dataset, only the subtrees with a cardinality between the 1st and the 3rd quartile (208.75 and 746.50 leaves, respectively) were retained. For each subtree, the leaves were extracted and the CNA matrix was generated; additionally, the position of their roots within the parental tree was tracked. From now on, we refer to this scenario as the *100Kcells* experiment.

The same procedure was executed to generate trees with 10K leaves, tracking subtrees for the first 20 generated cells. Also in this case, only the datasets with a cardinality between the 1st and the 3rd quartile (318 and 623.75 leaves, respectively) were kept. From now on, we refer to this scenario as the *10Kcells* experiment.

#### SHscore and MRCA distance correlation

In order to investigate the correlation between the SHscore and the distance between the MRCAs of the sample cells, we used the dataset generated in the *100Kcells* experiment. First, we computed the SHscores for the 4950 possible pairs of samples. After that, we randomly sampled 1000 pairs and computed the distance between their MRCAs, represented by the number of edges connecting the single cells that originated the two subtrees. We verified that the random selection was representative of the whole set of pairs (Supplementary Material: Supplementary Figure 2).

**Fig. 7:**
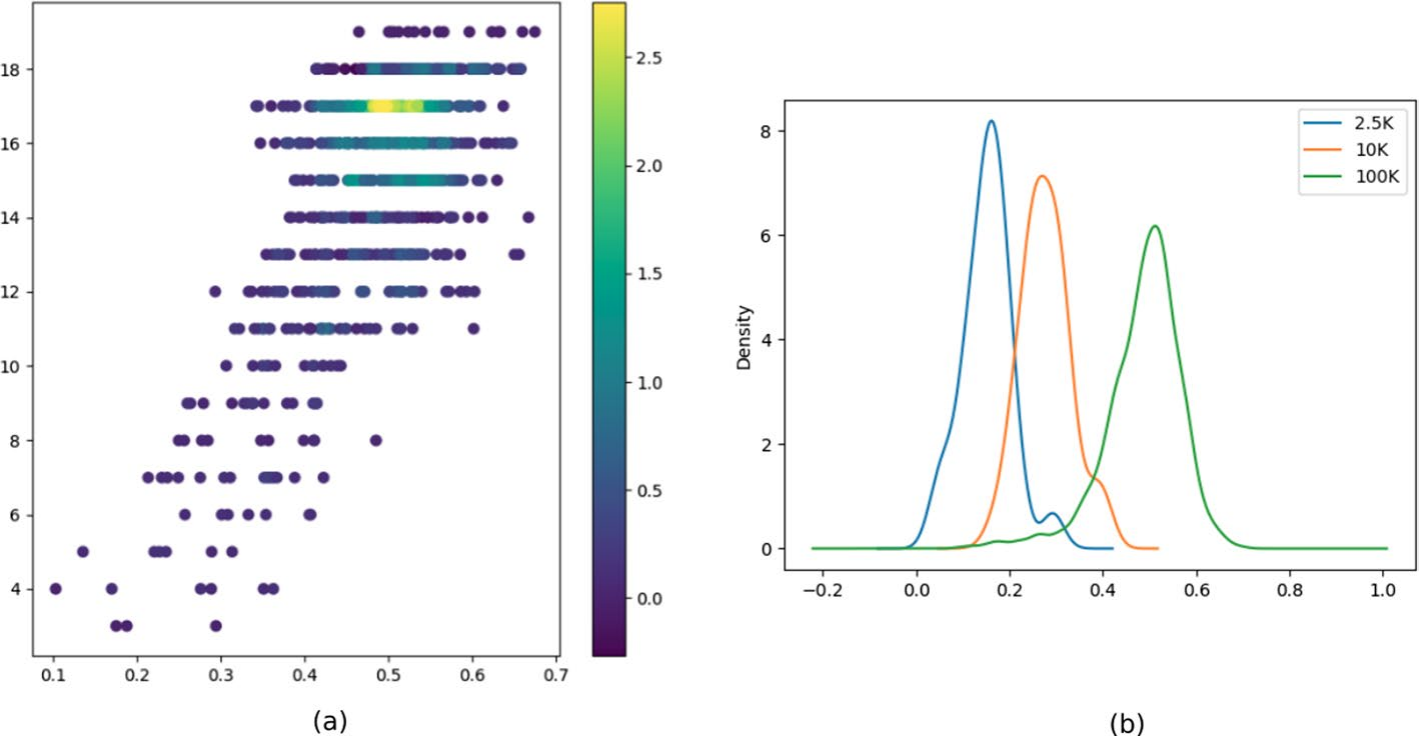
SHscore versus evolutionary distance. We executed a Pearson correlation test on the SHscores and the MRCA distances, demonstrating that the two quantities are positively correlated (coef = 0.628, p value = 1e−11) (**a**). We aggregated the SHscores computed on datasets deriving from from trees which growth was stopped at a different height. The scores in the three scenarios are distributed around a different median (2.5 K cells = median: 0.151, IQR: 0.064, 10 K cells = median: 0.278, IQR: 0.061, 100 K cells = median: 0.498, IQR: 0.092), which value increases as the mean distance between the MRCAs of the sample increases (**b**)

Finally, we were able to demonstrate that the two quantities are positively correlated, with a Pearson correlation coefficient c = 0.628 (p value = 1e−11, Fig. 7a).

This result verified the hypothesis that the heterogeneity measured by the SHscore captures the evolutionary distance of the cells belonging to the samples analyzed.

#### SHscore for different evolutionary spans

We computed the SHscores between the 45 pairs of samples generated from the *10Kcells* experiment and combined the results with those obtained in the *hom_scenario* and in the *100Kcells* experiment. The samples in the three scenarios contain a comparable number of cells ($\sim$ 500) but derive from trees which growth was stopped at a different height. This means that the sample history, in the three scenarios, diverged at different heights on the parental tree and kept on growing for a comparable number of doublings, at the same mutation rate, which is fixed by the generating model. Therefore, sample cells, in the three different scenarios, are likely to have accumulated the same amount of heterogeneity, starting from their MRCAs, while their divergence is mainly due to the heterogeneity accumulated by their MRCAs, which are located at different distances on the parental tree (very close on 2.5K cell trees, very distant on the 100K cell tree, intermediate distance on the 10K cell tree). Figure 7b shows that the scores in the three scenarios are distributed around a different median (2.5*K* cells = median: 0.151, IQR: 0.064, 10K cells = median: 0.278, IQR: 0.061, 100K cells = median: 0.498, IQR: 0.092), which value increases as the mean distance between the MRCAs of the sample increases.

This is an additional proof of what was shown before: the closer the MRCAs, the higher the score.

The results shown in this section lead us to conclude that a score lower than 0.2 indicates that the subclones are well-mixed in the tumor sample or that they are segregated in space, but spatial differences are so small that the tumor may be considered homogeneous. A score greater or equal to 0.2, instead suggests that different regions of the same tumors are separated by a non-negligible evolutionary distance which made them quite different and this should be considered for eventual further analyses.

### Experiment 3: SHscore on tumor data

Here, we present three examples of application of PhyliCS on real scCNA public datasets.

#### Spatial subsamples from the same disease site

This example shows how PhyliCS may be used to investigate spatial intra-tumor heterogeneity at a single disease site.

We have used PhyliCS on five single-cell CNA datasets produced with Cell Ranger DNA and published on 10x Genomics website [53]. The datasets derive from five sections (S_A, S_B, S_C, S_D, S_E), of the same frozen breast tumor tissue and contain data related to 2137, 2224, 1722, 1916 and 2053 cells, respectively.

*scCNA calling* We performed a few preliminary steps to produce PhyliCS input files. Specifically, we demultiplexed 10x multi-cell alignment files to get single-cell .bam files, using a C++ based tool, *SCtools*, we developed with the SeqAn library [54]. After that we performed some quality checks and computed CNA events using Ginkgo [34]. At this point, we were ready to load scCNA datasets into PhyliCS.

*Data Pre-processing* Using the preprocessing module, we removed diploid or pseudo-diploid cells (ploidy ranging in the interval [1.6, 2.9]), which are uninformative, and those which CNA profile was characterized by a high (>95th percentile) median absolute deviation (MAD), because they are considered noisy, due to single cell amplification issues or ongoing DNA replication. As a result, the cells left for the five samples were 110, 1172, 1040, 1137 and 1473. Since S_A contained very few tumor cells compared to the other samples, we did not include it in the following analysis steps.

**Fig. 8:**
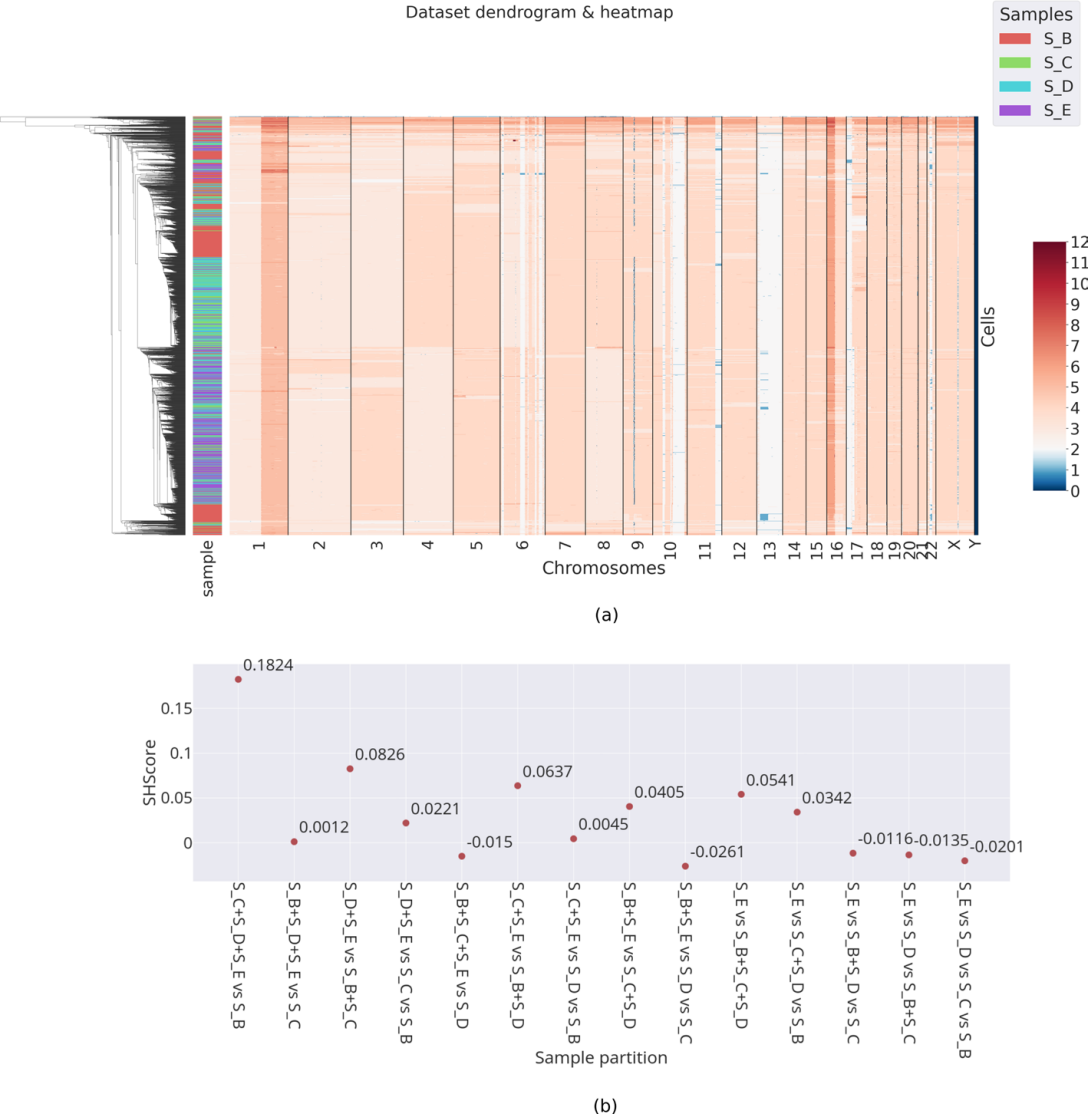
Test case: breast tumor data. We tested PhyliCS on a scCNA dataset containing the data of five sections (S_A, S_B, S_C, S_D, S_E) of the same breast tumor. After some preliminary operations, we discarded S_A and kept the others for further analysis. We obtained the evidence that the sections share similar genomic patterns (**a**), with the exception of S_B; this is confirmed by the SHscore (**b**), which best value (0.1824) is obtained by by aggregating S_C, S_D, S_E against S_B

*Multi-Sample Analysis* Figure 8a shows the graphical results produced after the aggregation phase. The cells from the four samples share a similar CNA profile and have been mixed-up by the clustering algorithm.

Figure 8b, instead, presents a diagram containing the SHscores computed for different sample aggregations. The value indicated as ’S_B vs S_C vs S_D vs S_E’ indicates how much the samples are different from each other. According to what we have seen with the simulation experiment, the value −0.0201 indicates that the four samples show a very similar genomic make-up, which makes them almost indistinguishable. Additionally, it can be noticed that by combining the samples S_C, S_D and S_E and testing them against S_B, the SHscore grows to $\texttt {0.182388}$, indicating that its genomic make-up may be clonally separated from that of the other samples. SHscores confirm the graphical results shown in Fig. 8a, highlighting S_B as the more divergent sample, a results that is backed up by the clonal reconstruction made by CHISEL [39], which reveals a subclone (J-I) that is almost private to that sample.

#### Spatial subsamples from the different disease sites

We also applied our method to a pair of samples derived from a primary tumor and a matched metastasis. To do this, we exploited the results of the CNA analysis performed by Garvin et al. on a dataset to validate Ginkgo [34]. The dataset corresponds to a primary breast tumor and its liver metastasis (T16P/M) and was used by Navin et al. [55] for their study on intra-tumor heterogeneity characterization. Since the CNA calls were available on Ginkgo website, we were able to directly load data into PhyliCS.

*Data Pre-processing* Also in this case, we filtered out diploid and pseudo-diploid cells and those with a high MAD, reducing the aggregated dataset cardinality from 100 to 42 cells.

**Fig. 9:**
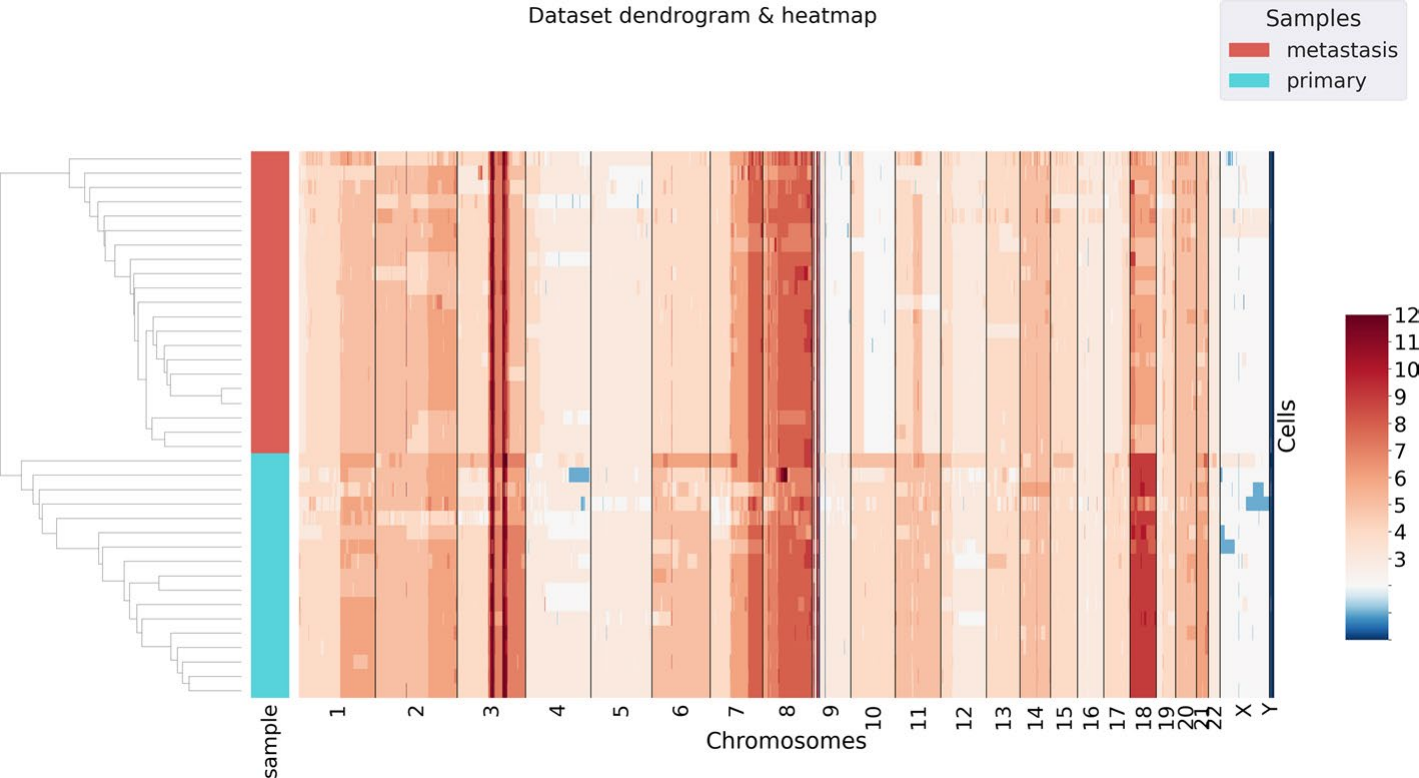
Test case: lung tumor data. We tested PhyliCS on pair of samples derived from a primary lung tumor and a matched liver metastasis. This time, the two samples shown a certain degree of genetic diversity and where characterized by a high SHscore (0.5361)

*Multi-Sample Analysis* Figure 9 presents the results obtained from the analysis performed on this dataset. It shows that, apparently, the same cell-population which initiated the tumor also seeded the metastasis, confirming the findings of the original publication [55]. The hierarchical clustering algorithm, this time, has organized cells in two separate blocks, corresponding to the two populations from the primary tumor and the metastasis. This underlines a certain degree of separation between the two samples, that is also represented by the SHscore. Even if we cannot compare scores for different sample arrangements, the SHscore (0.5361) is consistent with the results we obtained on metastatic scenarios simulations. The high SHscore means that although the primary and metastatic sample share a common mutational pattern, their following, independent, evolution made them clearly distinguishable. This suggests that the differences between primary and metastatic pairs that have always been measured with bulk sequencing can be further studied with scDNA approaches [56, 57].

#### Clonal expansion of a cell line

This example presents an extended use-case which shows how PhyliCS may be used to investigate the heterogeneity gained by a clonally expanded cell line.

In details, we exploited a single-cell dataset, recently published by Minussi et al. [58] on NCBI Sequence Read Archive (accession number PRJNA629885), containing the sequencing reads of cells from a triple-negative breast cancer cell line (MDA-MB-231) (508 cells) and those resulting from the clonal expansion of 2 single daughter cells (MDA231-EX1 and MDA231-EX2) from the parental cell line for 19 cell doublings (995 and 897 cells, respectively). From the sequencing reads, aligned to the GRCh38 reference genome, we called the CNA events using Ginkgo [34] (additional details on the alignment and CNA calling procedures are available at Supplementary Material: Supplementary Method 1).

**Fig. 10:**
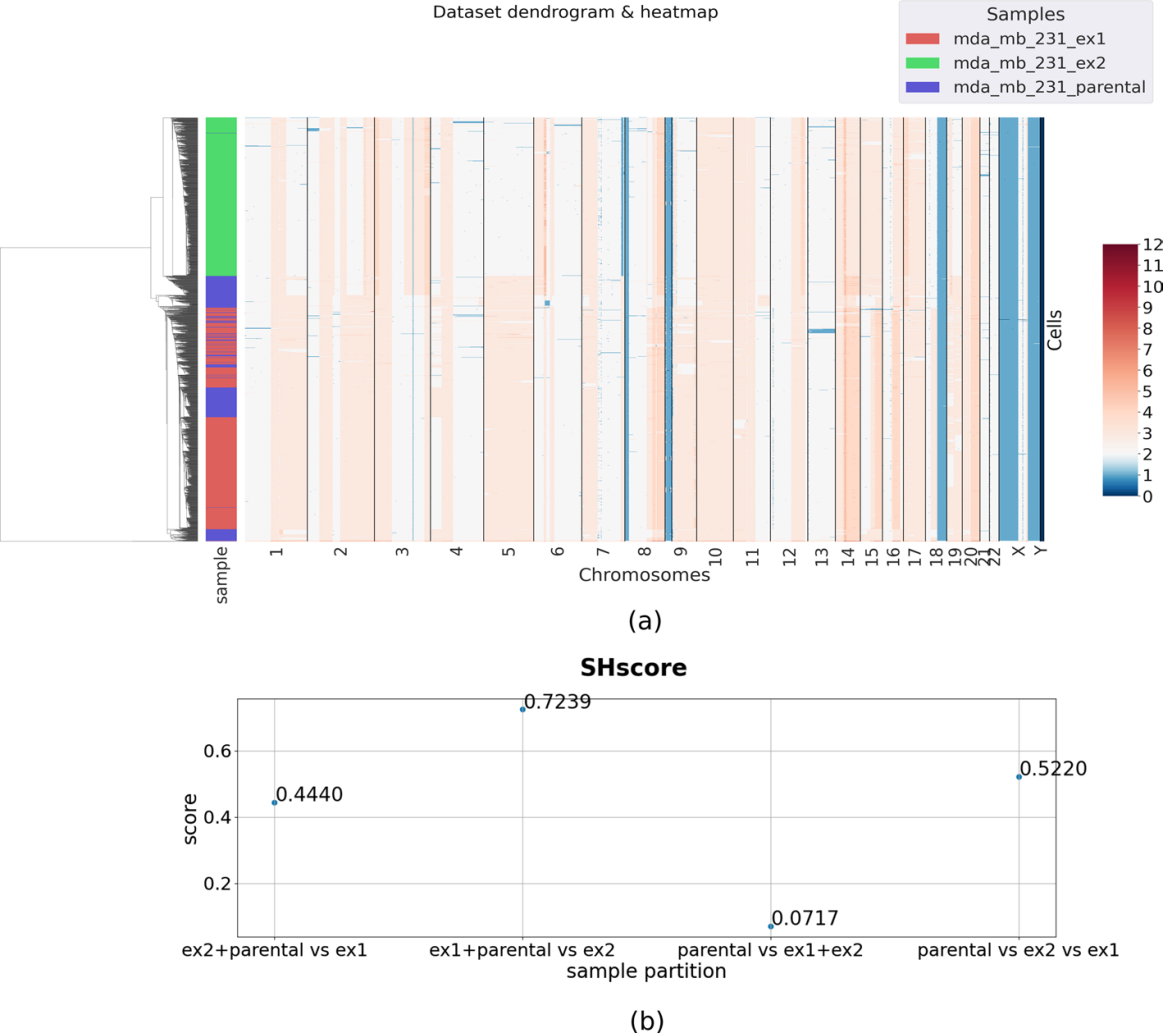
Test case: MDA-MB-231 cell line data. We tested PhyliCS on MDA-MB-231 cell line. In details, we compared the parental cell-line the the datasets resulting from the clonal expansion of two dauther cells, MDA-MB-231-EX1 and MDA-MB-231-EX2, for 19 doublings. The datasets contain 508, 995 and 897 cells respectively. We obtained the evidence that the dataset deriving from the expansion of MDA-MB-231-EX1 was more similar to the parental line, with respect to the genomic profile of the data deriving from MDA-MB-231-EX2 (**a**). In fact, the best SHscore (0.7102) was obtained when aggregating MDA-MB-231-EX1 dataset with the parental against the other one (**b**)

*Multi-Sample Analysis* We provided CNA matrices to PhyliCS and computed the SHscores for all possible partitions of the three datasets. Figure 10b shows that the best SHscore (0.7102) was obtained when aggregating MDA-MB-231-EX1 dataset with the parental one. This result indicates that MDA-MB-231-EX1 cells share a common genomic pattern with the parental cell line. This is confirmed by the results of the hierarchical clustering performed on the aggregated dataset, graphically shown in Fig. 10a: cells from MDA-MB-231-EX1 are well mixed with the parental ones, while the cells from MDA-MB-231-EX2 are put into a completely separate block. This may due to two reasons: the clonal expansion from MDA-MB-231-EX2 originating cell generated more heterogeneity than the other one or the clonal subpopulation which MDA-MB-231-EX2 originating cell was sampled from is not represented in the parental dataset (Supplementary Material: Supplementary Method 1). Anyhow, we can state that the proposed score is capable of capturing the different levels of diversity among multiple samples and when using it in a comparative way it is highly informative.

We exploited this dataset to present other PhyliCS features, analyzing separately the parental and derived cell lines. In particular we were able to demonstrate that the SHscore is robust when comparing two samples with different number of cells; specifically the heterogeneity measured between the derived cell lines does not significantly change when sampling different fractions of cells for the two samples (Supplementary Material: Supplementary Methods 2 and 3, Supplementary Figures 3, 4, 5 and 6).

## Conclusion

In this work we presented PhyliCS, a flexible and user-friendly package which allows to process scCNA calls and evaluate spatial ITH through the Spatial Heterogeneity Score. This score combines the high resolution of scDNA sequencing data and the information provided by multi-regional sampling to indicate how much different sets of cells have diverged in their CN landscapes, allowing to get fast and easy-to-interpret information about a single tumor.

PhyliCS has been implemented as a modular and flexible Python library, with many functionalities, which guides bioinformaticians who want to explore their datasets to use a single API specific for scDNA and tailored to its analysis.

We have tested the SHscore in different scenarios. First, we computed it on 200 synthetic datasets to study its behaviour in four different scenarios (spatial segregation, spatial intermixing, early metastasis spreading and late metastasis spreading). Results obtained on this set of simulations show that SHscore correctly reflects the heterogeneity in the clonal composition of multiple samples, and can therefore be used to reliably compare the heterogeneity of real tumors with different spatial samplings available. After that, we tested the SHscore on a set of 100 simulations, which were generated by randomly varying the mean CNA size and the mean number of gained copies, and found out that the score is not correlated to such structural features of the CN profiles. We conducted a more extensive simulation experiment, generating two big cell-division trees, to produce datasets with a significant evolutionary history. We got evidence that the SHscore is strongly correlated to the distance between the copy-number states which generated the cells of the samples in analysis. This confirmed that the SHscore captures the evolutionary history of the tumor subsamples. We used our score to analyze three real scDNA datasets, reaching conclusions in agreement with state of the art phylogenetic approaches [39] and the original papers [55, 58] that presented them. Finally we conducted a downsampling experiment on two cell line data to demonstrate that the SHscore is robust to sample cardinality and may be used in on unbalanced sets.

We have also demonstrated some of the analytical functionalities of the library, which allow the user to seamlessly perform tasks, which would generally require using different libraries and managing data flow between them.

We believe that trying to define clinically relevant thresholds for the SHscore is premature. Indeed, large cohorts of clinically annotated single-cell datasets, from patients affected by different tumors, would be required to correlate the evolutionary features of each tumor with its clinical characteristics and subsequently define thresholds to discriminate between “spatially segregated” and “spatially well-mixed” scenarios of of clinical relevance. Unfortunately, such single-cell DNA datasets are not yet available. However, from our extended simulation study, we got the evidence that a score lower than 0.2 indicates that the subclones are well-mixed in the tumor sample or that they are segregated in space, but spatial differences are so small that the tumor may be considered homogeneous. A score greater or equal to 0.2, instead suggests that different regions of the same tumors are separated by a non-negligible evolutionary distance which made them quite different and this should be considered for eventual further analyses.

One of the current limitations of PhyliCS is that all its results regarding evolutionary distances are derived from samples relationships and clustering based metrics. We opted for this approach in order to draw conclusions that, albeit simplistic, are based on less assumptions on the mechanisms driving CN accumulation, than the ones needed to perform phylogenetic reconstruction. Being the infinite site assumption not valid for CNs we think that phylogenetic reconstruction is still an open issue for single cell data; but we foresee that in the future there will be more reliable methods to call SNVs on single cells, opening new avenues to exploit the theoretical knowledge built upon bulk sequencing.

In summary, PhyliCS represents a valuable instrument to explore the extent of spatial heterogeneity in multi-regional tumour sampling, exploiting the potential of scCNA data.

In the future, scDNA sequencing should gain popularity, and more data will be available on public repositories; at that point, we would like to test and improve our score on large scale datasets. Additionally, it will be interesting to integrate different single cell measurements, such as ATACseq or scRNA, to extend its capabilities. The choice to develop a library should ease future endeavours in this direction.

## Availability and requirements

Project name: PhyliCS

Project home page: https://github.com/bioinformatics-polito/PhyliCS

Operating systems: GNU/Linux, MacOS and Windows

Programming language: Python

Other requirements: gcc to install HDBSCAN Python library

Licence: GNU Affero General Public License v3 (AGPL3)

Any restrictions to use by non-academics: None

## Supplementary Information

**Additional file 1.** Supplementary Material containing the supplementary figures and methods cited in the main text.

## Data Availability

PhyliCS is distributed via PyPI (https://pypi.org/project/phylics/) and Bioconda (https://anaconda.org/bioconda/phylics). Its source code and a minimal documentation are available on GitHub: https://github.com/bioinformatics-polito/PhyliCS. Data and results discussed in the paper are all stored in a dedicated repository and summarized by means of jupyter notebooks accessible through: https://github.com/bioinformatics-polito/PhyliCS_usage. The datasets used in benchmarks have been obtained by simulations. The datasets used in demostrations are publicly available on 10x Genomics (https://support.10xgenomics.com/single-cell-dna/datasets), Ginkgo (http://qb.cshl.edu/ginkgo/?q=/igjlK8I6pGAWvGWeqS9P) websites and on NCBI Sequence Read Archive(PRJNA629885). SCtools source code is available on Github at https://github.com/bioinformatics-polito/SCTools.

## Abbreviations

ITH: Intra-tumor heterogeneity
scDNA: Single-cell DNA
CNA: Copy-number aberration
scCNA: Single-cell copy-number aberration
scDNA-seq: Single-cell DNA sequencing
SHscore: Spatial-Heterogeneity score
