# Supplementary material for: PhyliCS: a Python library to explore scCNA data and quantify spatial tumor heterogeneity
Source: BMC Bioinformatics. 2021 Jul 3;22:360. doi: 10.1186/s12859-021-04277-3 (PMC8254361; doi:10.1186/s12859-021-04277-3)
Supplement: Supplementary file 1 — Additional file 1. Supplementary Material containing the supplementary figures and methods cited in the main text. [file 12859_2021_4277_MOESM1_ESM.pdf]

PhyliCS: a Python library to explore scCNA data and quantify spatial tumor heterogeneity Supplementary Material

Marilisa Montemurro, Elena Grassi, Carmelo Gabriele Pizzino, Andrea Bertotti, Elisa Ficarra and Gianvito Urgese

*marilisa.montemurro@polito.it

1. Supplementary Figures

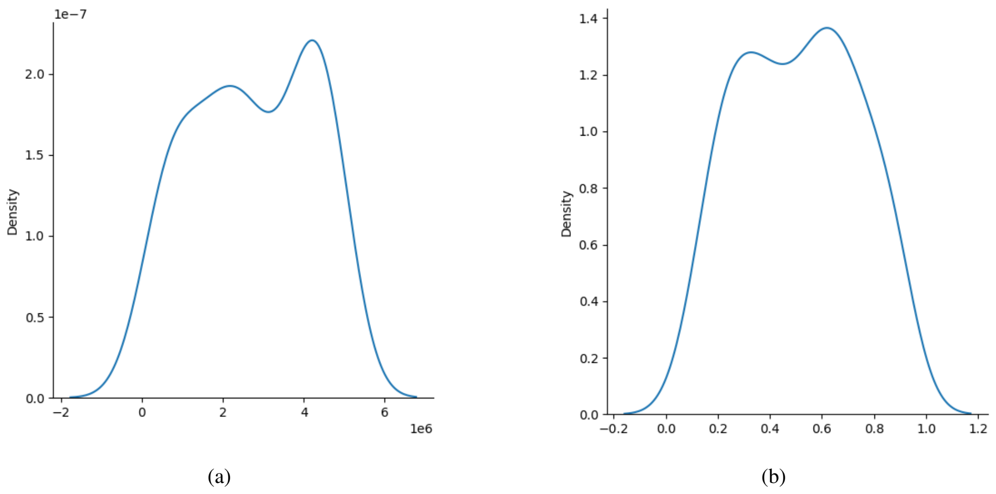

Fig. 1. **Supplementary Figure 1**: distribution of the values randomly sampled for the parameters 1a and p 1b used to seed the simulations in the var-scenario (Experiment 1: SHscore on synthetic data. Simulations with varying parameters.)

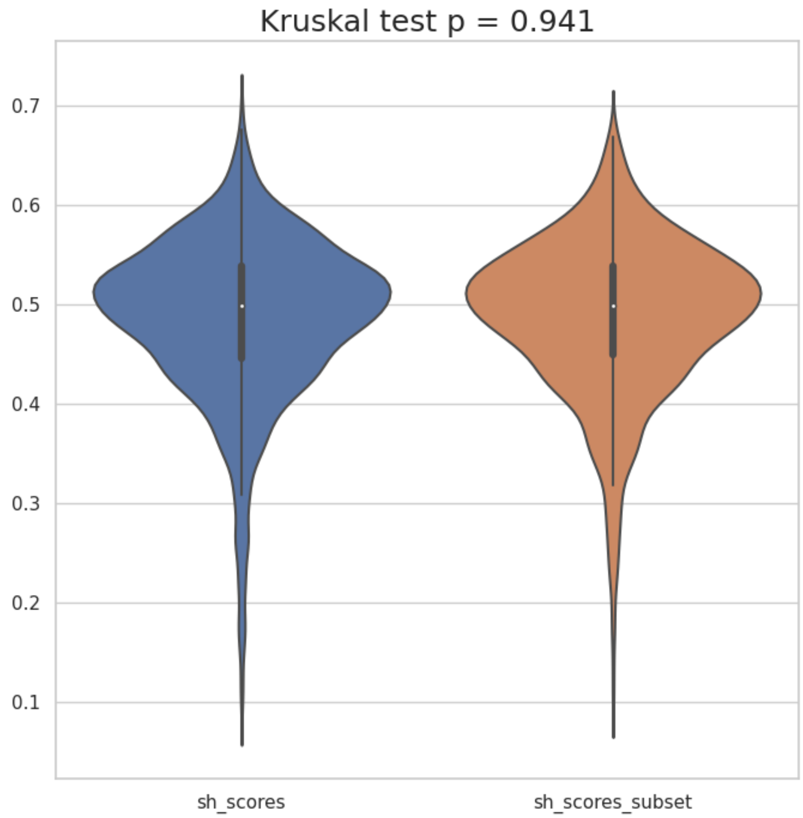

Fig. 2. **Supplementary Figure 2**: comparison distribution of the full set of SHscores computed for the 4950 pairs of samples and the distribution of the 1000 randomly sampled. The two set of scores are equally distributed (Kruskal-Wallis pvalue = 0.941), so the SHscore subset is representative of the full set of scores (Experiment 2: SHscore and evolutionary distance. SHscore and MRCA distance correlation).

2. Supplementary Methods

Supplementary Method 1: sequencing reads processing and scCNA calling

We downloaded, from the NCBI Sequence Read Archive (accesion number PRJNA629885), the sequencing reads of cells from a triple-negative breast cancer (TNBC) cell line (MDA-MB-231) and those resulting from the clonal expansion of 2 single daughter cells (MDA231-EX1 and MDA231-EX2) from the parental cell line for 19 cell doublings (995 and 897 cells, respectively). Concerning the parental dataset, two batches of cells where available: 508 obtained with single-end sequencing and 312 resulting from paired-end sequencing. To avoid a batch effect, we only download the cells sequenced using the single-end technique. We aligned the single cell reads against the GRCh38 reference genome using BWA (v0.7.17) [1]. We filtered out low quality reads (MAPQ < 20), secondary alignments and PCR duplicates using SAMtools (v1.9) [2]. After that, we produced the BED files using BEDTools (v2.27.1) [3]. We, finally, computed the CNA

events, for the three datasets, separately, using a standalone version of Ginkgo [4], with variable binning (mean bin size = 500kb) and default options. To generate boundaries for variable-length bins, for the reference genome, we used the method outlined by Garvin et al. [4] and implemented at Ginkgo repository: basically, it consists in sampling 101bp reads from the reference genome and mapping it back to itself (BWA), looking for uniquely mapping reads. After that, for the given bin size, reads are assinged to bins such that each bin has the same number of uniquely mappable reads. Consequently, intervals with higher repeat contents and low mappability will be larger than intervals with highly mappable sequences, although they will both have the same number of uniquely mappable positions.

Supplementary Method 2: MDA-MB-231 clustering

We furtherly investigated the MDA-MB-231 dataset with PhyliCS.

Data prepocessing

First of all, we performed dimensionality reduction with the UMAP method provided by PhyliCS, to improve scCNA data clustering performance [5]. Then, we chose the optimal number of clusters, using `nk_clust()` method, which computes clustering quality according to multiple indices (Silhouette coefficient, Calinski and Harabasz score or Davies-Bouldin score), for a given clustering algorithm and a specified range of k's. For the current use-case, the Silhouette Coefficient was computed to evaluate Agglomerative Clustering performance (ward linkage), with min k = 3 and max k = 10. As Supplementary Table 1 shows, the best coefficient was obtained with k=3.

**k**	**silhouette**
**3**	0.729
**4**	0.593
**5**	0.652
**6**	0.671
**7**	0.700
**8**	0.601
**9**	0.571
**10**	0.531

Table 1. **Supplementary Table 1**: Silhouette Coefficients computed on the results of Agglomerative Clustering, with min_k = 3 and max_k = 10, applied to MDA-MB-231 parental cell line.

Clustering

As Supplementary Figures 3a and 3b show, the clusters obtained with k = 3 are coherent with the distribution of the data. However, it is clear that cluster 0 may still be divided into subgroups: thus, we clustered data with k = 7, the second optimal choice according to the Silhouette index, and obtained a more refined clustering of the data (Supplementary Figures 4a and 4b).

It can be noticed, in Figure 4b, that the two prevalent cluster of cells, clusters 3 and 4, are characterized by a CN profile very similar to that of MDA-MB-231-EX1 cells, confirming the results found during the multisample analysis.

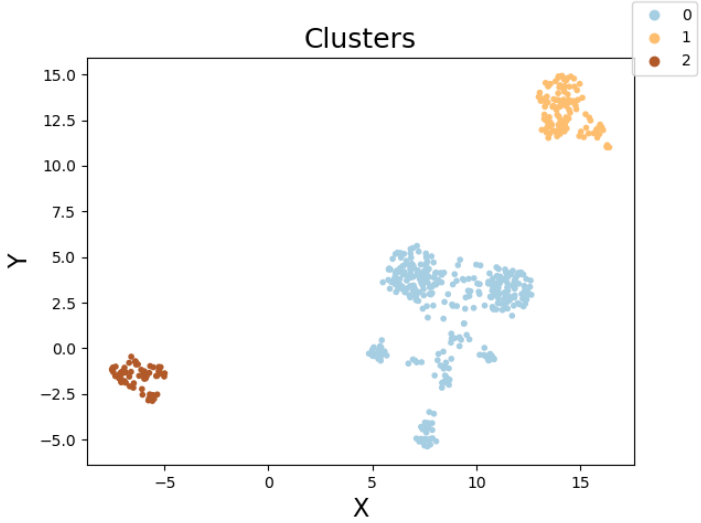

(a)

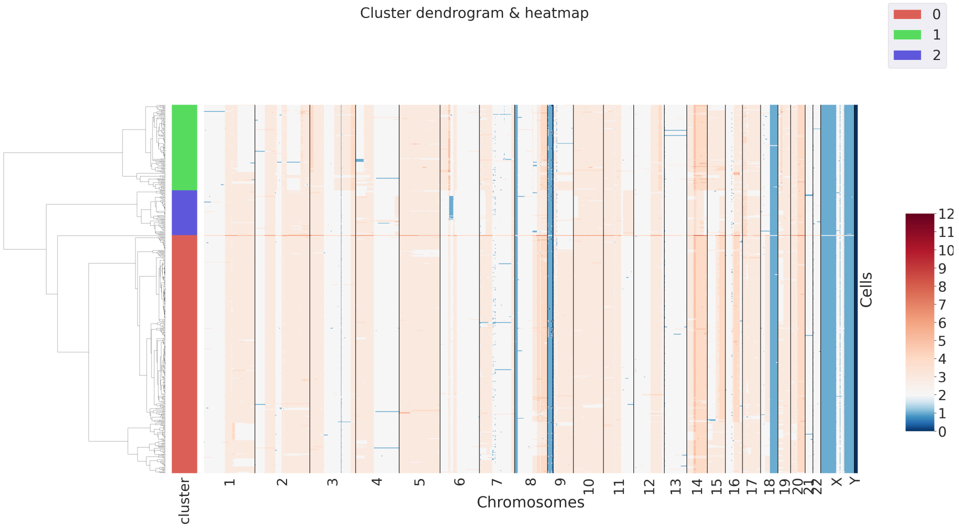

(b)

Fig. 3. **Supplementary Figure 3**: Agglomerative Hierarchical Clustering results with k = 3. 3a. 2D projection of clusters (UMAP embeddings). 3b. CN profiles organized into a dendrogram. (Experiment 3: SHscore on tumor data. Clonal expansion of a cell line.)

Supplementary Method 3: SHscore robustness to dataset cardinality

In a real world scenario, the number of cells sequenced may vary from sample to sample. For this reason, we executed multiple downsampling experiments on the daughter cell lines to test the robustness of the SHscore to the cardinality of the samples

The two cell lines are characterized by a comparable number of cells (995, 897) and derive from a clonal expansion of two single cells. For this reason, they are expected to be internally genetically homogeneous and some random subsamples should not be consistently different

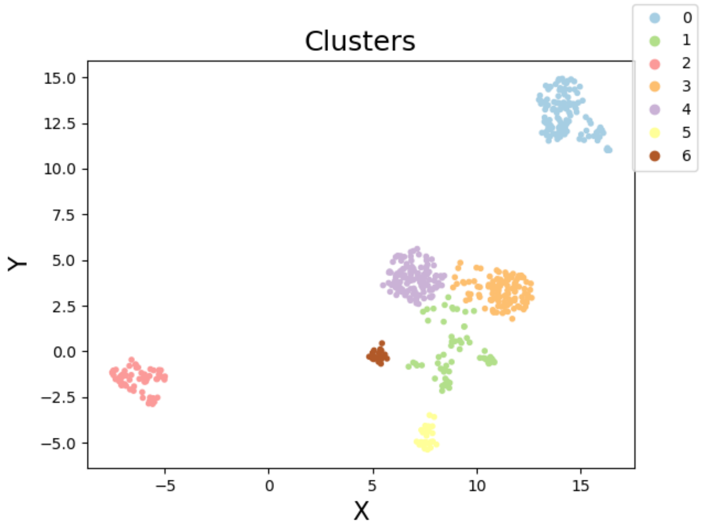

(a)

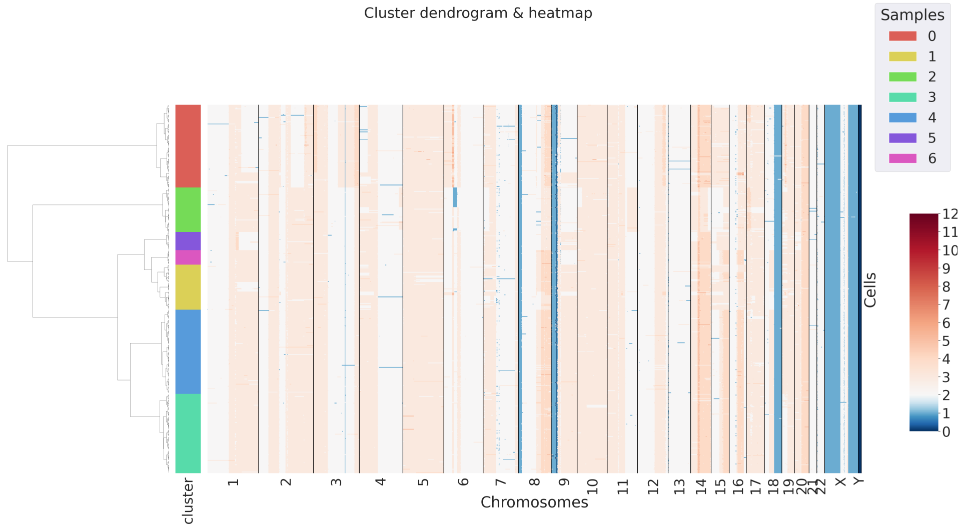

(b)

Fig. 4. **Supplementary Figure 4**: Agglomerative Hierarchical Clustering results with k = 7. 4a. 2D projection of clusters (UMAP embeddings). 4b. CN profiles organized into a dendrogram. (Experiment 3: SHscore on tumor data. Clonal expansion of a cell line.)

among each other. Consequently, when comparing two subsamples, with varying cardinalities, from the two cell lines, we expect the SHscore to remain, almost, stable.aa

As first step, we computed the SHscore using full datasets. Supplementary Figure 5 shows that our hypothesis of internal genetic homogeneity is confirmed while the SHscore value, 0.832, indicates that the two cell lines are characterized by well distinguishable CN profiles.

After that, we generated 9 subsamples from each cell line by randomly selecting a fraction (10%, 20%, 30%, 40%, 50%, 60%, 70%, 80%, 90%) of its cells. We computed the SHscore on all pairs made of one of the two full datasets (e.g MDA-MB-231-EX1) and one of the subsamples from the

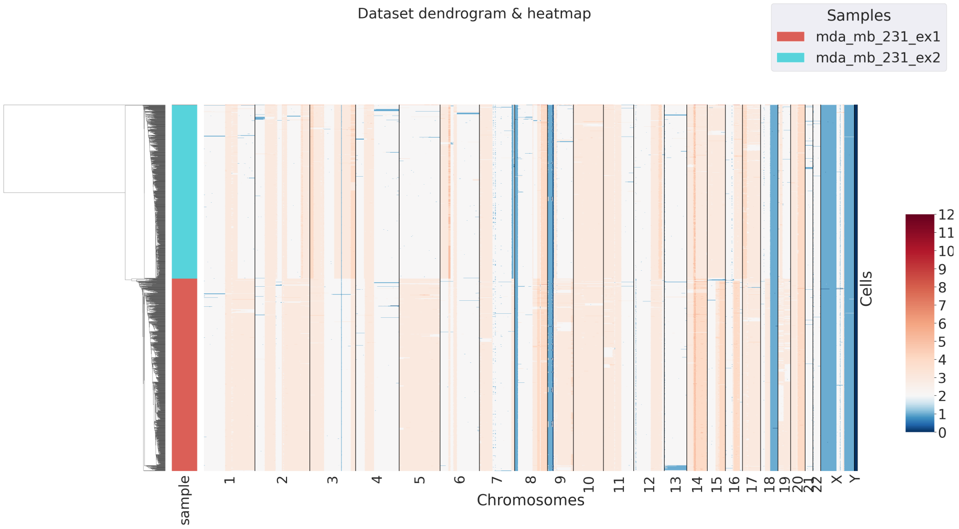

Fig. 5. **Supplementary Figure 5**: MDA-MB-231-EX1 vs MDA-MB-231-EX2. We performed multi-sample analysis on the two daughter cell lines. The hierarchical clustering algorithm separated their cells into two well-separated and internally homogeneous blocks. The SHscore (0.832106) confirmed this evidence.

other one (e.g. MDA-MB-231-EX2_10%, MDA-MB-231-EX2_20%, etc.). As Supplementary Figure 6 shows, the resulting SHscores fluctuated of a small quantity with respect to the initial SHscore. Specifically, they where distributed in the interval [0.815, 0.850], with median = 0.833 and IQR = 0.015. The small fluctuations of the score should not surprise because, albeit being internally homogeneous, the two cell lines still present a subclonal structure [6], so the downsampling operation may have targeted cells belonging to different subclones. Anyhow, the fact that the median result is almost identical to the original SHscore indicates that the proposed method is robust to the cardinality of the datasets and may be used to compare samples of any size; additionally, it demonstrates that the SHscore is able to capture heterogeneity even when the input dataset cardinalities are very unbalanced.

References

1. H. Li and R. Durbin, "Fast and accurate short read alignment with burrows–wheeler transform," bioinformatics **25**, 1754–1760 (2009).
2. P. Danecek, J. K. Bonfield, J. Liddle, J. Marshall, V. Ohan, M. O. Pollard, A. Whitwham, T. Keane, S. A. McCarthy, R. M. Davies *et al.*, "Twelve years of samtools and bcftools," GigaScience **10**, giab008 (2021).
3. A. R. Quinlan and I. M. Hall, "Bedtools: a flexible suite of utilities for comparing genomic features," Bioinformatics **26**, 841–842 (2010).
4. T. Garvin, R. Aboukhalil, J. Kendall, T. Baslan, G. S. Atwal, J. Hicks, M. Wigler, and M. C. Schatz, "Interactive analysis and assessment of single-cell copy-number variations," Nat. methods **12**, 1058–1060 (2015).
5. M. M. Montemurro, G. G. Urgese, E. G. E. Grassi, C. G. CGP, A. A. Bertotti, and E. E. Ficarra, "Effective evaluation of clustering algorithms on single-cell cna data," in *2020 7th International Conference on Biomedical and Bioinformatics Engineering,* (2020), pp. 5–11.
6. D. C. Minussi, M. D. Nicholson, H. Ye, A. Davis, K. Wang, T. Baker, M. Tarabichi, E. Sei, H. Du, M. Rabbani *et al.*, "Breast tumours maintain a reservoir of subclonal diversity during expansion," Nature **592**, 302–308 (2021).

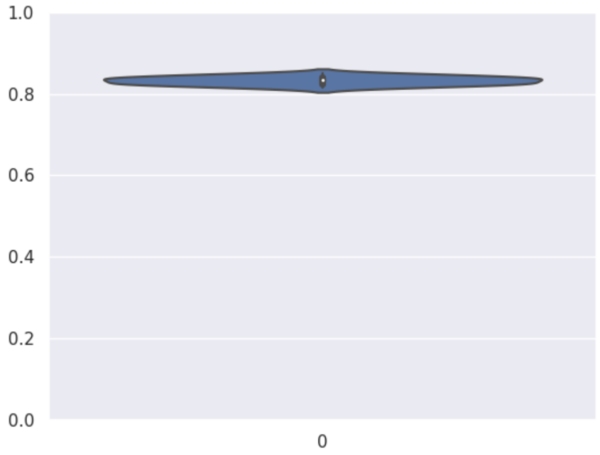

Fig. 6. **Supplementary Figure 6**: Downsampling experiment. In order to test the robustness of the proposed method we downsampled both daughter cell lines, producing 9 subsamples for both of them, each containing a fraction 10%, 20%, 30%, 40%, 50%, 60%, 70%, 80%, 90% of their cells. We computed the SHscore on pairs made of one of the two full datasets against each of the subsamples of the other dataset and observed their distribution: despite small fluctuations, due to the little amount of heterogeneity existing in the the cell lines, the computed SHscores (min = 0.815, max = 0.850, median = 0.833, IQR = 0.015) were comparable to that computed for the orginal dataset (0.832), confirming the proposed method is robust to the cardinality of the input datasets.
